# YjgA plays dual roles in enhancing PTC maturation

**DOI:** 10.1093/nar/gkae469

**Published:** 2024-06-06

**Authors:** Mengtan Du, Chenke Deng, Ting Yu, Qixin Zhou, Fuxing Zeng

**Affiliations:** Department of Systems Biology, School of Life Sciences, Southern University of Science and Technology, No. 1088 Xueyuan Avenue, Shenzhen 518055, People's Republic of China; Institute for Biological Electron Microscopy, Southern University of Science and Technology, No. 1088 Xueyuan Avenue, Shenzhen 518055 Guangdong, People's Republic of China; Department of Systems Biology, School of Life Sciences, Southern University of Science and Technology, No. 1088 Xueyuan Avenue, Shenzhen 518055, People's Republic of China; Department of Systems Biology, School of Life Sciences, Southern University of Science and Technology, No. 1088 Xueyuan Avenue, Shenzhen 518055, People's Republic of China; Institute for Biological Electron Microscopy, Southern University of Science and Technology, No. 1088 Xueyuan Avenue, Shenzhen 518055 Guangdong, People's Republic of China; Department of Systems Biology, School of Life Sciences, Southern University of Science and Technology, No. 1088 Xueyuan Avenue, Shenzhen 518055, People's Republic of China; Institute for Biological Electron Microscopy, Southern University of Science and Technology, No. 1088 Xueyuan Avenue, Shenzhen 518055 Guangdong, People's Republic of China; Department of Systems Biology, School of Life Sciences, Southern University of Science and Technology, No. 1088 Xueyuan Avenue, Shenzhen 518055, People's Republic of China; Institute for Biological Electron Microscopy, Southern University of Science and Technology, No. 1088 Xueyuan Avenue, Shenzhen 518055 Guangdong, People's Republic of China

## Abstract

Ribosome biogenesis is a highly regulated cellular process that involves the control of numerous assembly factors. The small protein YjgA has been reported to play a role in the late stages of 50S assembly. However, the precise molecular mechanism underlying its function remains unclear. In this study, cryo-electron microscopy (cryo-EM) structures revealed that depletion of YjgA or its N-terminal loop in *Escherichia coli* both lead to the accumulation of immature 50S particles with structural abnormalities mainly in peptidyl transferase center (PTC) and H68/69 region. CryoDRGN analysis uncovered 8 and 6 distinct conformations of pre50S for Δ*yjgA* and YjgA-ΔNloop, respectively. These conformations highlighted the role of the N-terminal loop of YjgA in integrating uL16 and stabilizing H89 in PTC, which was further verified by the pull-down assays of YjgA and its mutants with uL16. Together with the function of undocking H68 through the binding of its C-terminal CTLH-like domain to the base of the L1 stalk, YjgA facilitates the maturation of PTC. This study identified critical domains of YjgA contributing to 50S assembly efficiency, providing a comprehensive understanding of the dual roles of YjgA in accelerating ribosome biogenesis and expanding our knowledge of the intricate processes governing cellular protein synthesis.

## Graphical Abstract

**Graphical Abstract:**
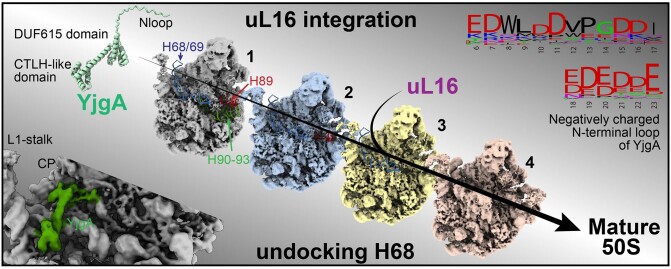

## Introduction

The ribosome is a vital organelle responsible for protein synthesis in all living organisms. Disruption of protein synthesis leads to well-documented defects in cell growth and cell cycle control that are, in turn, translated to various disease states (1). In prokaryotes, the ribosome comprises a small 30S subunit and a large 50S subunit that assemble independently (2). Compared to the 30S subunit, our understanding of the *in vivo* assembly of the 50S subunit is more limited. This is attributed to the greater complexity and intricate folding of the rRNA within the 50S subunit (3).

To study the structural aspects of ribosome biogenesis factors in bacterial systems, researchers have utilized knockout strains targeting specific biogenesis factors (such as RbgA, SrmB and RrmJ) (3–5) or ribosomal proteins (bL17, uL16) (3,6) to obtain intermediate structures (3,6–8). Additionally, knockin strains of *Escherichia coli* with tagged *obgE* inserted at its gene locus have been employed to visualize native 50S precursors (pre50S) bound with ObgE (7). Other approaches, such as incubating the isolated mature 50S subunits or pre50S with purified factors, e.g. EngA (8) or RbgA (9), serve as initial attempts to address the binding sites of these biogenesis factors in pre50S intermediates. Based on their functions, ribosome biogenesis factors can be categorized into two main groups: RNA-modifying enzymes and RNA-remodeling proteins (10). RNA-modifying enzymes primarily act on rRNA after transcription, adding specific chemical modifications to the rRNA molecule, thus altering the chemical properties of the bases or ribose (2,11,12). The disruption of rRNA modification could result in abnormal assembly and function of ribosomes. For example, the absence of methyl-transferase RrmJ causes severe growth defects and marked accumulation of pre50S intermediates (13). Additionally, the folding of rRNA and the interaction between ribosomal proteins and rRNAs rely on the assistance of RNA remodeling proteins, such as RNA helicases, chaperones, and GTPases (14–16). However, further improvements are needed in understanding the molecular mechanisms through which these factors monitor and influence the ribosomal assembly process, as well as in obtaining a comprehensive picture of the interactions among these constituents. Additionally, the presence of many identified but functionally unknown or unidentified proteins in the process of 50S assembly still impedes our understanding of this process (2).

YjgA initially has been identified as a protein co-migrating with 50S particles in the CgtAE/ObgE mutant strain through mass spectrometry (10). Subsequent structural investigations on Δ*bL17*, tagged-ObgE and *in vitro* assembled pre50S complexes revealed that YjgA selectively interacted with pre50S (6,7,17). The pre50S-associated YjgA shows two distinct conformations: one presents a dumbbell-liked shape surrounded by bL31 and H74/80/93 (6,18,19), which connects the base of the central protuberance (CP) and the lower part of L1-stalk. In contrast, the other conformation connects the base of the CP with the top part of the L1-stalk, which is bent and moves away from its neutral position toward the CP (6,7). Based on these observations, a model proposing the YjgA-dependent pathway of 50S assembly was put forward, suggesting that YjgA undergoes dynamic changes and influences the conformation of H68/69 (6,7). However, this model falls short of elucidating the molecular mechanism of YjgA in the 50S assembly process. H68 is a long helix lying in the middle of the 50S surface and the anchor of H68 is located at the edge of PTC. The two short helices, H69 and H71, form the other side of the anchor. The connecting point between H68/H69 and PTC is H71. It has been reported that during *in vitro* assembly, H71 mediates the proper folding of H90–H93 within PTC (Supplemental Figure S1) (2). In the studies of pre50S intermediates previously reported, two states have been observed for H68. One is the ‘docked’ state, in which H68 is similar to the one in mature 50S and usually shows good density. The second is the ‘undocked’ state, in which H68 is invisible, and H69/71 also exhibits high flexibility. In addition, YjgA is part of a tightly regulated network, including ObgE, RluD, RsfS, etc. They contributed to the processing of the pre50S subunit, specifically involving the folding of H89 within the peptidyl transfer center (PTC) (7). PTC is the ancient catalytic core of the ribosome and, possibly as a consequence, free of r-proteins that could aid RNA folding (20). In modern ribosomes, it spans from H89 to H93 and interacts with the CCA-ends of the A-site and P-site tRNAs, thus directly participating in the peptide elongation and release reactions (20). *In vitro* experiments have demonstrated that the assembly of PTC is time-consuming without the assistance of assembly factors (21). Similarly, *in vivo* experiments show significant impacts on PTC maturation when some assembly factors, e.g. ObgE and YjgA, are lacking (7,19). Although many such factors are identified, their precise model of action remains largely elusive, with crucial evidence for their role in assembly often derived from gene deletion experiments (22). Given the proximity of YjgA to H68 and the PTC, there is a growing interest in elucidating the precise function of YjgA in 50S assembly. Understanding the mechanism of action of YjgA would represent a significant advancement in unraveling the origin and characteristics of the PTC.

Here, we present a series of cryo-electron microscopy (cryo-EM) structures of pre50S intermediates in both Δ*yjgA* strain and the cells expressing a YjgA mutant with the N-terminal loop truncated (YjgA-ΔNloop). Structural analysis of pre50S_Δ_*_yjgA_* and pre50S_ΔNloop_ and 30S/pre50S profiling of the mutants suggested that YjgA plays a pivotal role in facilitating 50S subunit maturation through two primary mechanisms. First, by binding to the base of the L1-stalk with its C-terminal CTLH-like domain, YjgA inhibits the docking of H68, which results in a more flexible H71 and provides more space for PTC, thus accelerating the maturation of PTC. Second, the N-terminal loop of YjgA might interact with uL16, thereby promoting the binding of uL16 and resulting in a stabilization of H89, which further contributes to PTC maturation. Mutagenesis assay and pull-down assay further revealed that the negatively charged residues, aspartic acid (D), glutamic acid (E), and a hydrophobic residue I17 within this N-terminal loop, were responsible for the interaction. In summary, our study elucidated how YjgA regulates the assembly dynamics of H68/69 and H89, providing valuable insights into the intricate mechanisms governing ribosome maturation.

## Materials and methods

### Conservation analysis

To compare the YjgA from different species, the 143 reviewed YjgA sequences were collected from UniProt (23) and aligned with ClusterW. Conservation of the amino acids was shown by Weblog (24) or ConSurf (25).

### Strain and plasmids construction

Gene depletion of *yjgA* in *Escherichia coli* BL21 strain (Δ*yjgA*) was carried out by ubigene (https://www.ubigene.com/). To construct the plasmids for protein expression, the sequence of *yjgA* gene (NC_000913.3) and uL16 (NC_000913.3) were first obtained by PCR from the genome of *E.coli* BL21 and then subcloned into the expression vector pET28a and pET28a-GST (no his tag) using the EcoR I and Hind III restriction sites. The final constructs were verified by sequencing. For truncations including YjgA-ΔC (1–110 aa), YjgA-ΔN (111–183 aa), YjgA-ΔNloop (25–183 aa), YjgA-Δα5 (Δ105–116 aa), pET28a-YjgA-FL was used as PCR template and truncation were introduced in respected primers. For YjgA-Nloop mutants, pET28a-YjgA-FL was used as the PCR template, and truncation was introduced in respected primers.

### 30S/pre50S sucrose gradient profiles

YjgA-FL and the mutant constructs were transformed into Δ*yjgA* cells and grew at 37°C until the stationary phase. The pre-cultures were diluted in fresh LB medium to OD_600_ equaled to 0.05 and cultured to OD_600_ of 0.6. The expression of the proteins was induced by 0.05 mM IPTG at 16°C for 9h. The wild-type BL21 and Δ*yjgA* cells were also cultured in LB medium at 37°C until OD_600_ reached 0.6 and transformed into the 16 °C for 9 h as the same condition used for truncations expression assays. Cells were collected by centrifugation and lysed in a bead beater by tissueLyser (65W, 180s on ice in between) in lysis buffer (20 mM HEPES, pH 7.5, 150 mM NH_4_Cl, 10 mM MgCl_2_, 1 mM dithiothreitol). Cell lysates were clarified by centrifugation at 15 000 rpm for 30 min. The concentration of cleared lysates was adjusted to the same level, and 15 units of *A*_260_ were loaded on 10–50% sucrose gradients in lysis buffer. Ultracentrifugation was carried out in the SW40-Ti rotor (Beckman) at 38 000 rpm for 5 h at 4°C. The distribution of ribosomal populations was analyzed using a homemade system combined with AKTA.

### Spot assay

To analyze growth on solid media, *E. coli* cells were grown in LB medium at 37°C to exponential phase and diluted to a cell density of OD_600_ was 0.3. Serial dilutions with a dilution factor of 10 were prepared and transferred to LB agar plates. Plates were incubated at 16°C and 37°C until single colonies became visible.

### Cryo-EM sample and grid preparation

Based on the UV 260 nm trace, gradient fractions corresponding to the pre50S ribosome peak of Δ*yjgA* and YjgA-ΔNloop were collected separately. For EM analysis, the combined fraction was diluted to 4 units of *A*_260_ in lysis buffer and buffer change 5 times using a 100 kDa cutoff concentrator to remove most of the sucrose. The concentration of the pre50S was estimated by *A*_260_.

An aliquot of 2.5 μl of freshly isolated pre50S_Δ_*_yjgA_* and pre50S_ΔNloop_ were applied to carbon grids with 2 nm continuous carbon film and cryo-plunged into liquid ethane after blotting using a Vitrobot device (FEI) operated at 4°C, 100% humidity with 3 s blotting time and 30 s incubation time.

### cryo-EM data collection and image processing

Movies were collected on a Titan Krios G3i, operated at 300 kV, with a Gatan K3 Summit camera. Data acquisition was performed using software EPU, with a nominal magnification of 81 000×, which yields a final pixel size of 1.05 Å at object scale (defocus ranging from –1.5 μm to –2.5 μm). For each micrograph stack, 30 frames were collected, with a total dose of 30 electrons per pixel.

For pre50S_Δ_*_yjgA_*, a total of 1126 micrographs were obtained, the motion was corrected using motioncor software (26), and the contrast transfer function (CTF) was estimated using CTFFIND-4.1 (27) in Relion (28). Relion 3.1.0 was also used for all subsequent particle picking, classifications, and refinement. Corrected and aligned micrographs were first subjected to auto-picking in Relion, resulting in a total of 350 446 selected particles. Then selected particles were extracted with a box size of 256 pixels, and submitted for two rounds of 2D classification. After removing the bad particles, the pre50S were classified into six classes using an initial 3D model from PDB (4YBB). The best three classes (pre50S particles) from 3D classification were selected and their particles were re-extracted with a box size of 384 pixels. A total of 205 861 particles was finally used for 3D auto-refinement, resulting in an overall resolution of 3.23 Å after postprocessing.

For pre50S_ΔNloop_, a total of 1307 micrographs were obtained and processed similarly to the pre50S_Δ_*_yjgA_*. A number of 174, 541 particles was finally used for 3D auto-refinement, resulting in an overall resolution of 3.63 Å after postprocessing.

### Model refinement

A high-resolution cryo-EM structure of the *E. coil* ribosome (PDB ID: 7K00) was rigid-body fitted into the consensus map using UCSF Chimera version 1.16 (29). In the final deposited model, all nucleotides not supported by the density were removed. No water oxygens or ions were modeled.

### Heterogeneous reconstruction by deep neural network

CryoDRGN (30) models were trained on the 205 861 particles obtained from the refined 3D structure of the pre50S_Δ_*_yjgA_* and 174, 541 particles of pre50S_ΔNloop_. Particle orientations, translations (poses) and CTFs were parsed from their assignments as part of the above 3D refinement. The original image dimensions 384 were directly down-sampled by Fourier cropping to 256 to optimally benefit from faster mix-precision training (by using a multiple of 8 box size). Then images were trained for 50 epochs using a 256 × 3 (nodes per layer × layers) architecture for both the encoder and decoder networks. The latent space dimensionality |*Z*| was 8. Any junk images were filtered out by interactively selecting outliers in the UMAP projection of the latent embeddings, including over one subsequent training using a 1024 × 3 model for 100 epochs. The kept particles were then retrained using a 256 × 3 architecture for 50 epochs as this was sufficient for learning OBD flexibility. Representative density maps were obtained by partitioning the latent encodings into 20 regions via *k*-means clustering, with the volumes then generated from data points closest in Euclidean distance to the cluster centers. For pre50S_Δ_*_yjgA_* and pre50S_ΔNloop_, the distribution of particles among their respective datasets is shown in the supplementary table.

### Pull-down assay

The constructions, including YjgA-FL, YjgA-ΔNloop, YjgA-DE-A, YjgA-E23A, YjgA-D15A in pET28a-His, and GST-uL16 in pET28a-GST were transformed into Δ*yjgA* and grew overnight. The pre-cultures were diluted in fresh LB medium to OD_600_ equaled to 0.05 and cultured to OD_600_ of 0.6. IPTG was added to a final concentration of 0.05 mM and cultures were grown for an additional 9 hours at 16 °C prior to sedimentation. Cells were resuspended in lysis buffer (20 mM Tris–HCl pH 8, 500 mM NaCl, 10% glycerol) and disrupted by valve homogenizer at 4 °C. After disruption, the protein-containing lysate was centrifuged for 45 min at 18 000 rpm and 4 °C before filtration using a 0.45 mm PVDF filter membrane. Ni-NTA beads were equilibrated with pull-down buffer (20 mM Tris–HCl, pH 8.0, 150 mM NaCl, 10% glycerol), mixed with clarified cell lysate (YjgA-FL, YjgA-ΔNloo, YjgA-DE-A, YjgA-E23A and YjgA-D15A). The mixture was poured into a gravity column and flow through was collected by gravity flow. Beads were washed with 50 mL pull-down buffer with the addition of 25 mM imidazole and eluted with 20 ml pull-down buffer with the addition of 250 mM imidazole. GST-uL16 protein was purified with GST column which eluted by 15 mM reduced glutathione in pull-down buffer.

Bacterially expressed GST-uL16 (0.5mg) was immobilized on Glutathione Sepharose 4B and then incubated with YjgA and YjgA-Nloop mutants proteins (1 mg) in 1 ml pull-down buffer for 2 h at 4°C. The resin was washed three times with the same buffer, eluted by 15mM reduced glutathione in pull-down buffer and then analyzed by SDS-PAGE.

## Results

### Depletion of *yjgA* perturbs formation and maintenance of mature ribosomes

**Figure 1.**
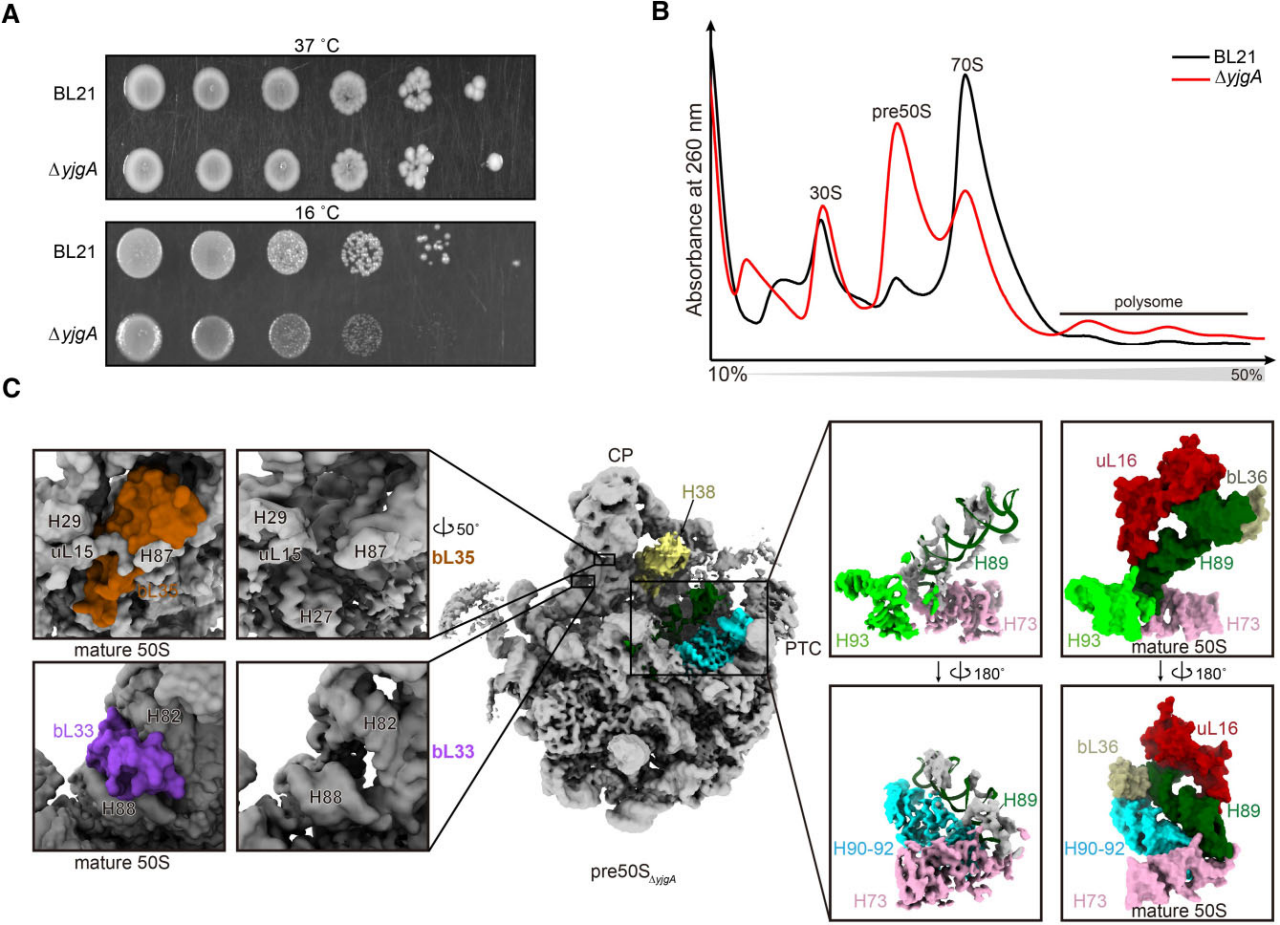
YjgA participates in the late stage of 50S maturation. (**A**) Spot assays of wild type BL21 *E. coli* strain and Δ*yjgA* strain at 37°C and 16°C. (**B**) 10–50% sucrose gradient profiles for BL21 (black) and Δ*yjgA* (red) strains. An accumulated pre50S peak is shown for the Δ*yjgA* strain. (**C**) Overall structure of the pre50S_Δ_*_yjgA_*. Details of the PTC, bL33 and bL35 are zoomed up to represent the density occupancy of H89, uL16, bL33, bL35 and bL36. The models of the PTC, bL33 and bL35 in a mature 50S (PDB: 7K00) are shown in the surface as a reference.

Here, to delve deeper into characterizing the functions of YjgA in 50S assembly, we conducted structural studies on pre50S intermediates in a *yjgA* depletion background (Δ*yjgA*). As expected, the Δ*yjgA* strain exhibited a growth rate similar to that of wild-type BL21 cells at 37°C, but it displayed slower growth at 16°C (Figure 1A), which is consistent with an assembly defect, as most mutations affecting ribosome assembly are cold-sensitive (31). When the cells were grown at 16°C, it also showed an accumulation of a pre50S peak in the sucrose gradient profiles at 10 mM Mg^2+^ condition (Figure 1B, red line). The presence of a low amount of mature 70S subunits shown in sucrose gradient profiles indicates that the formation of 50S is not completely blocked, which explains why the Δ*yjgA* stain can still grow at low temperatures, albeit slower than the wild-type (Figure 1A and B). To structurally characterize these pre50S intermediates, we collected the peak and subjected them to cryo-EM analysis (Figure 1C). As depicted in Supplemental Figure S2, the results of 3D classification indicate that the pre50S intermediates, referred to as pre50S_Δ_*_yjgA_*, constitute the majority (∼80%) of all the particles analyzed. Notably, mature 30S and 70S were contaminated from the near peaks, while no mature 50S subunits were observed within this population, suggesting the low amount of 50S subunits formed is rapidly incorporated into translating ribosomes and does not accumulate as free entities (Supplemental Figure S2). Analysis of the pre50S_Δ_*_yjgA_* intermediates revealed the absence of densities corresponding to several ribosomal proteins, including uL16, bL33, bL35 and bL36 (Figure 1C), bearing resemblance to the protein profiles observed *in vivo* from 40S or 45S particles isolated from various *E. coli* or *B. subtilis* strains with disrupted genes encoding different assembly factors, such as *csdA, srmB, rrmJ, dnaK, dbpA, engA, yphC, ysxC* and *obgE* (4,7,8,32–36). In addition to the perturbation of assembly factors, deletion of certain ribosomal protein genes, such as L5, L27 and L28, also induces the accumulation of *in vivo* intermediates lacking these proteins (37–39). For the rRNA, H38, L1 stalk and H89 showed weak density in the reconstruction (Figure 1C), indicating that the assembly of these late-stage proteins, including bL33 and bL35, and rRNAs containing H38 and L1 stalk, are susceptible to various forms of disruption. These structure changes reveal that deletion of *yjgA* only affects the late steps of 50S maturation.

### The immature particles of pre50S_Δ_*_yjgA_* exhibit multiple conformations

**Figure 2.**
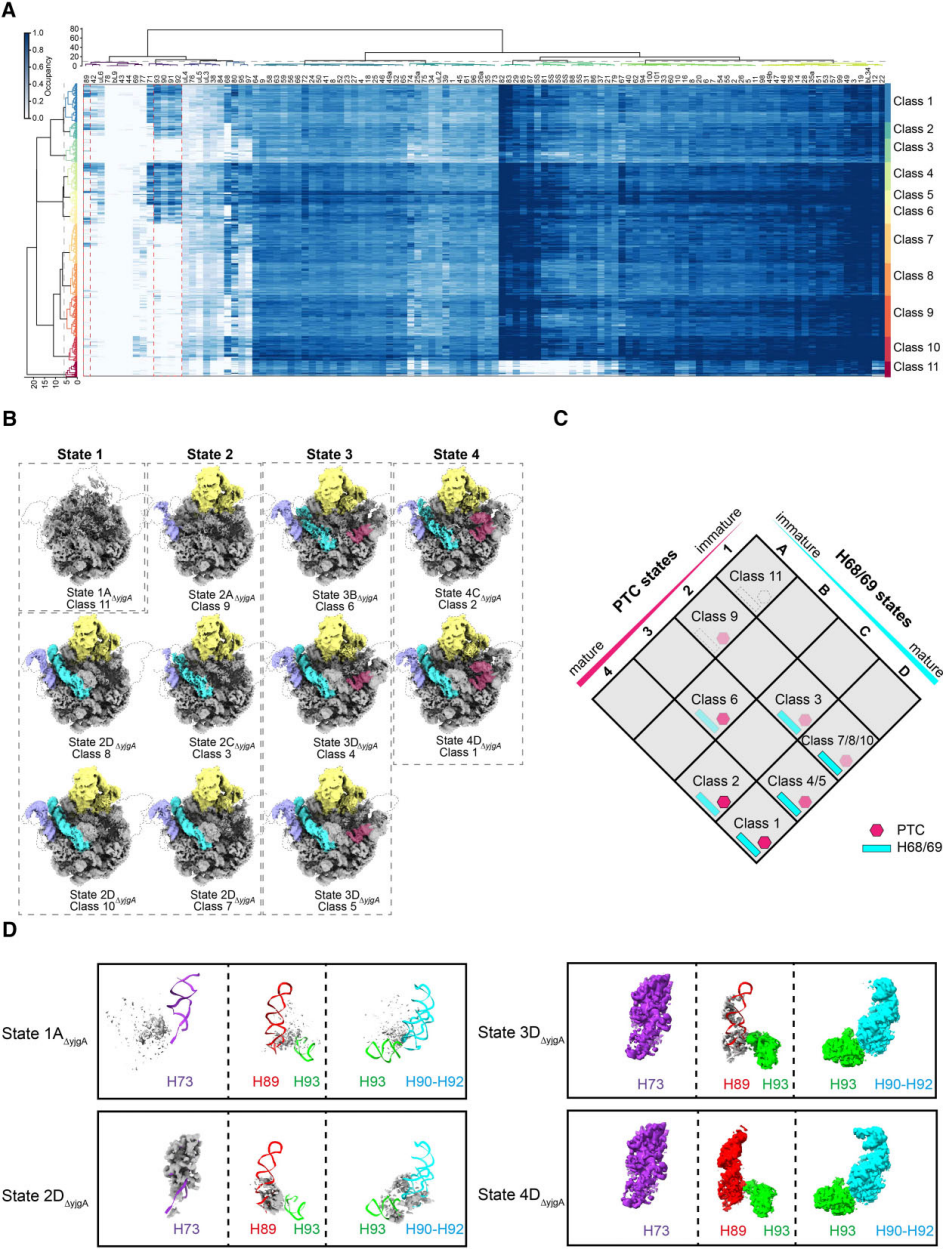
Pre50S_Δ_*_yjgA_* exhibits multiple conformations with structural variants in PTC and H68/69 region. (**A**) Five hundred volumes of pre50S_Δ_*_yjgA_* were segmented using cryoDRGN, and a heatmap was generated based on their similarities. Each color on the right represents one class. The intensity of the heat map reflects the degree of similarity between different volumes. (**B**) Eleven distinct density map groups were obtained by classification using cryoDRGN. Each image shows the average density map and reconstruction model of the corresponding group. These 11 maps represent 11 different structural states found in the sample. Of these, purple is L1 stalk, yellow is CP and pink is PTC. (**C**) Based on the maturation state of H68/69 and PTC, 11 density maps were classified into 8 distinct groups. These groups represent structural changes in pre50S at different stages of H68/69 and PTC maturation. States 1–4 and A–D correspond to pre50S intermediates with varying degrees of maturation. Blue represents the maturation of H68/69, and pink represents the maturation of PTC. (**D**) The densities of the PTC region are represented as a surface for the pre50S_Δ_*_yjgA_* states 1A_Δ_*_yjgA_*, 2D_Δ_*_yjgA_*, 3D_Δ_*_yjgA_* and 4D_Δ_*_yjgA_*. A model of a mature 50S (PDB: 7K00) is shown in the cartoon as a reference.

To gain a detailed understanding of the subunit assembly dynamics for pre50S_Δ_*_yjgA_* particles, we employed cryoDRGN, an advanced computational tool for cryo-EM data analysis (30). By directly taking the position of particles calculated in Relion, cryoDRGN grouped them into different classes using various clustering methods, such as *k*-means, PCA (principle component analysis), and UMAP (uniform manifold approximation and projection) (Supplemental Figure S3A). While, the best one came from the hierarchical clustering using Euclidean distance, and the linkage method was set to ‘ward’, which minimized the variance of the clusters being merged. In this study, all 205 861 particles were grouped into 500 volumes using *k*-means clustering with *K* = 500 so that each volume contains enough particles as reported previously (Supplemental Figure S3B) (30). Then, the coordinate of a mature 50S model was split into 114 blocks according to their positions in the 50S (30). After calculating the occupancy of these 114 pdb-blocks in each volume, these 500 volumes were then clustered into 11 classes (Figure 2A and Supplemental Figure S3C). This classification approach effectively resolved the sample heterogeneity, as illustrated by the 11 distinct classes obtained (Figure 2B).

These 11 classes display a series of structures for pre50S intermediates, from those with the most regions invisible to those resembled closely to the mature 50S (Figure 2B). The core region, which includes the core block and blocks of uL29, bL20, H72, uL2, uL14, uL15, bL28, H42 and base block as defined previously (17), is assembled in all 11 classes, whereas the CP block, PTC and H68/69 display different levels of folding (Figure 2B). Based on these features, the 11 classes were named state 1A_Δ_*_yjgA_* to 4D_Δ_*_yjgA_* accordingly, where the numbers 1 to 4 indicate the maturation status of PTC, and the letters A to D represent the formation of H68/69 (Figure 2B and C). State 1A_Δ_*_yjgA_* is the earliest intermediate obtained here, with only a core region folded, and all other states show a well-folded CP (Figure 2B). State 2A_Δ_*_yjgA_* have PTC and H68/69 unfolded, and only partial densities are observed around the PTC region (Figure 2C). From state 2 to state 4, the region of PTC shows a stepwise maturation (Figure 2C, magenta). As seen in state 2, H73 starts to fold, and the partial density shows a different base-pairing pattern for this helix (Figure 2D, State 2D_Δ_*_yjgA_*). State 3B_Δ_*_yjgA_* and 3D_Δ_*_yjgA_*have completely folded H73 and H90–93 but still show a flexible H89 (Figure 2D, State 3D_Δ_*_yjgA_*). While in state 4, all the helices in the PTC region display good densities, indicating PTC is fully matured in this state (Figure 2D, State 4D_Δ_*_yjgA_*). On the other hand, states B to D show a different level of density for the long helix H68/69 (Figure 2C). State 3B_Δ_*_yjgA_* has the weakest density for H68/69, indicating a high flexibility of this long helix (Figure 2C). Notably, H68/69 in states 3B_Δ_*_yjgA_*, 3D_Δ_*_yjgA_*, 4C_Δ_*_yjgA_* and 4D_Δ_*_yjgA_* show different occupancy as observed in states 2C_Δ_*_yjgA_* and 2D_Δ_*_yjgA_*, indicating a similar flexibility of H68/69 even when PTC is mature (Figure 2C). These distinct intermediates suggest that the absence of YjgA results in a diminished rate of 50S subunit assembly, primarily impacting H68/69 and PTC.

### The N-terminal loop of YjgA is important for the 50S assembly

**Figure 3.**
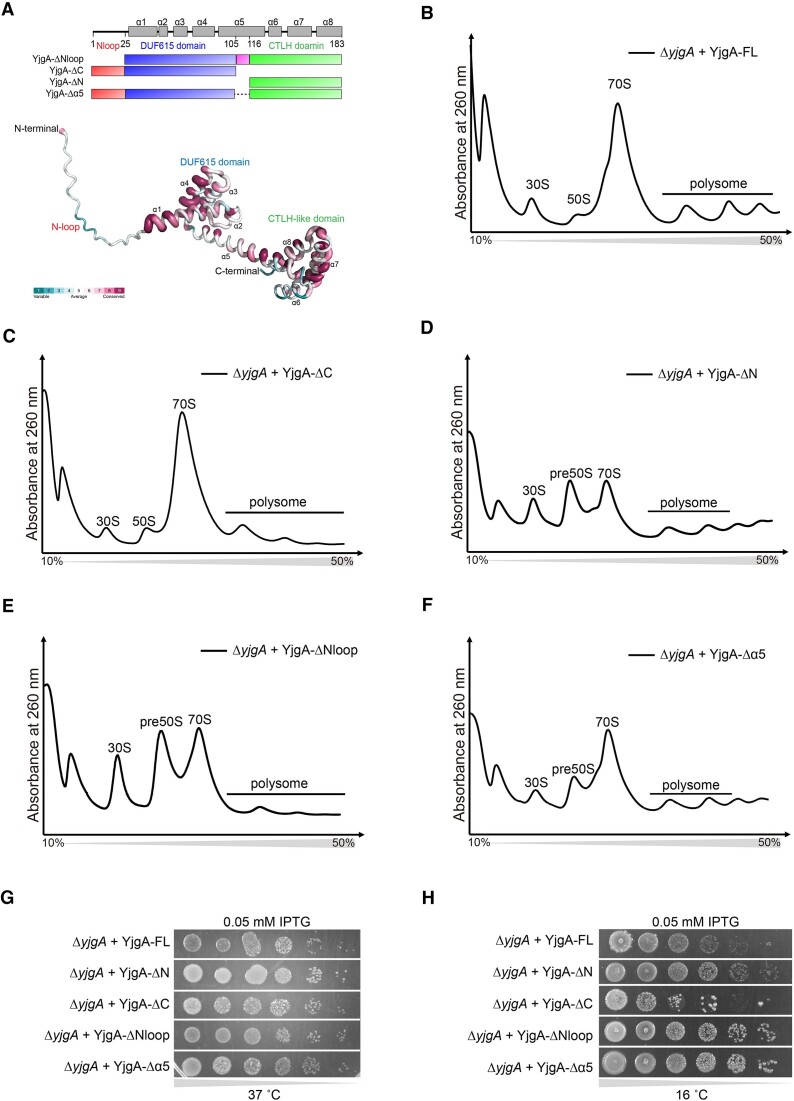
Different structure elements of YjgA function differently and cooperatively in 50S assembly. (**A**) Top: design of the YjgA truncations for functional studies. Bottom: the conservation of YjgA is shown in tubes representing the conservation scores along the structure. (B–F) Sucrose gradient profiles of cells harboring YjgA-FL (**B**), YjgA-ΔC (**C**), YjgA-ΔN (**D**), YjgA-ΔNloop (**E**) and YjgA-Δα5 (**F**) in the background of Δ*yjgA*. (G, H) Spot assay of cells harboring YjgA-FL and its mutants in the background of Δ*yjgA* at 37°C (**G**) and 16°C (**H**).

YjgA is a small conserved protein in bacteria that contains 183 amino acids, and its structure can be divided into four parts, including the N-terminal loop (Nloop), DUF615 domain, the long central alpha helix (α5), and the CTLH-like domain (Figure 3A). The DUF615 (domain of unknown function, PFAM accession number: PF04751) only exists in YjgA and its homologies. The CTLH-like domain (C-terminal to LisH) is also a predicted alpha-helical sequence of unknown function that is found adjacent to the LisH motif in several LisH proteins, contributing to the regulation of microtubule dynamics, either by mediating dimerization or else by binding cytoplasmic dynein heavy chain or microtubules directly (40). By comparing the 143 reviewed YjgA sequences adapted from UniProt, the major conserved regions were shown to be α1, α4 and α7 (Figure 3A and Supplemental Figure S4A). To investigate the importance of each element in YjgA, truncations were then constructed for the deletion of the N-terminal loop (YjgA-ΔNloop), keeping the N-terminal part (YjgA-ΔC) or C-terminal part (YjgA-ΔN), and shorten the long helix α5 (YjgA-Δα5) (Figure 3A). These truncations and full-length YjgA (YjgA-FL) were subsequently overexpressed in Δ*yjgA* cells at 37°C and 16 °C. Sucrose gradient analysis of YjgA-FL at 16°C overexpressed cells revealed a distribution of 30S, 50S, 70S, and polysome similar to that of BL21 cells, indicating overexpressed YjgA-FL functions normally in the Δ*yjgA* cells (Figure 3B compared to 1B). Overexpression of YjgA-ΔC and YjgA-ΔN at 16°C exhibited distinct profiles. YjgA-ΔC displayed a profile similar to YjgA-FL (Figure 3C), while YjgA-ΔN showed an accumulation of pre50S peak resembling Δ*yjgA* cells (Figure 3D). These findings indicate the critical role of the N-terminal region, and it is sufficient to rescue the function of YjgA-FL in pre50S assembly.

Although the N-terminal loop of YjgA was not observed in any YjgA/pre50S structures previously published, our results reveal that this loop is indispensable for the normal process of pre50S assembly. This is evident as deleting this loop resulted in the accumulation of pre50S peaks to the same level as in Δ*yjgA* and YjgA-ΔN profiles (Figure 3E). Shortening the helix that connects the N- and C-terminal parts (YjgA-Δα5) also resulted in moderate changes in the pre50S peak (Figure 3F), indicating that the proximity of the C-terminal to the N-terminal may affect the binding of YjgA-Δα5 to the pre50S or influence its conformational changes. All these results indicate that the structural elements of YjgA function differently but cooperatively.

Spot assays showed that Δ*yjgA* strain harboring YjgA-FL plasmid grows slower than BL21 strain with empty vector at 37°C and 16°C which means that YjgA-FL exerts inhibitory effects on bacterial growth (Supplemental Figure S4B). In addition, Δ*yjgA* harboring different overexpression plasmids exhibited similar growth rates as YjgA-FL at 37°C (Figure 3G). However, when the cells grow at 16°C, expression of YjgA-ΔN, YjgA-ΔNloop, and YjgA-Δα5 resulted in faster growth rates than YjgA-FL and YjgA-ΔC (Figure 3H). This is probably because the N-terminal domain especially Nloop exhibits strong inhibitory effects on bacterial growth, while the conserved C-terminal CTLH-like domain can reduce its inhibitory effects. Nevertheless, these results suggest that the growth rate in spot assay could not fully reflect the affection of YjgA on pre50S maturation.

### Mutation of the negatively charged residues in the N-terminal loop of YjgA impairs the 50S assembly

**Figure 4.**
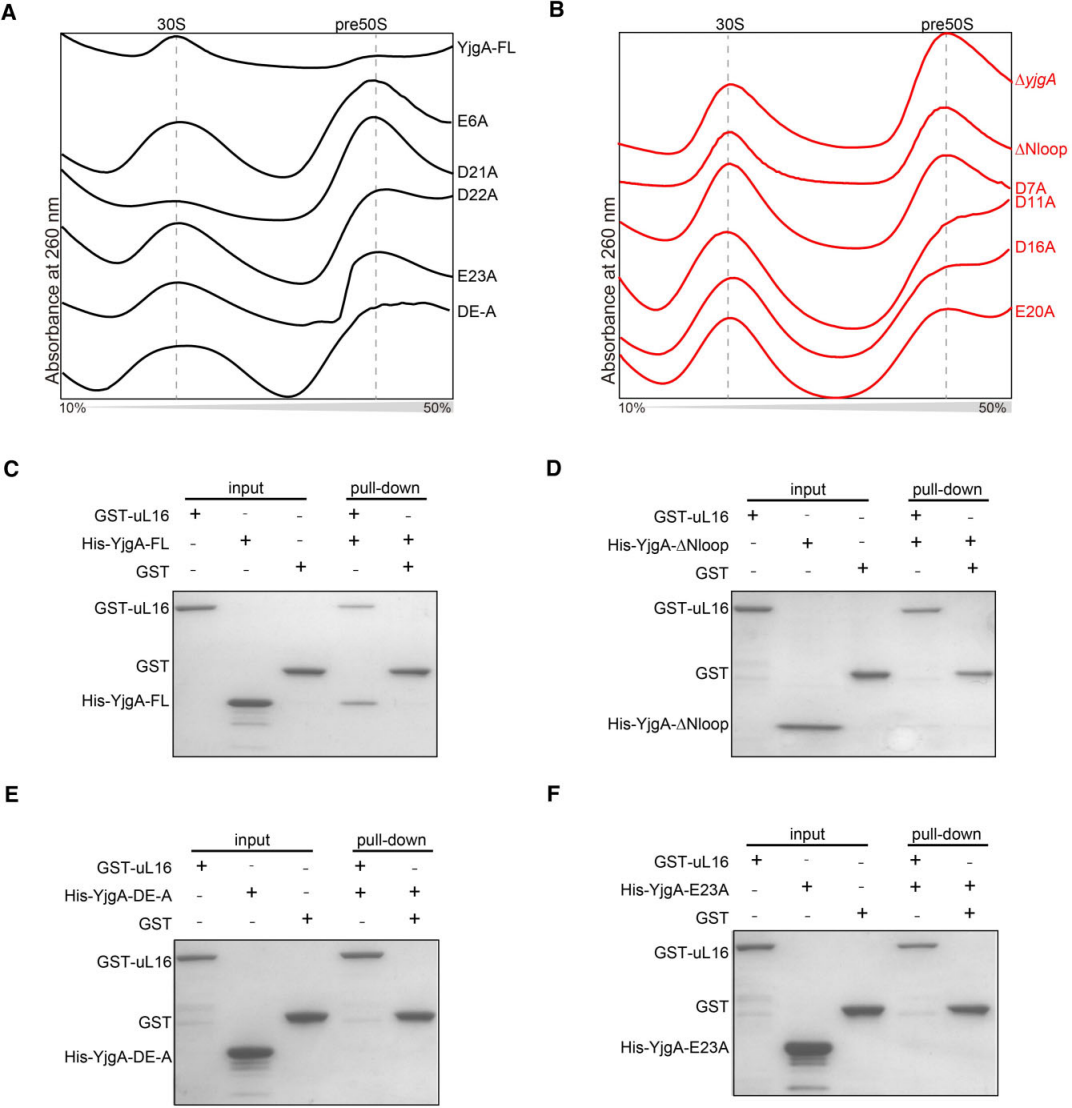
The N-terminal loop of YjgA is important for the 50S assembly. (**A**) Sucrose gradient profiles of cells harboring YjgA-FL and mutations, including E6A, D21A, D22A, E23A and DE-A in the background of Δ*yjgA*. Only 30S and 50S peaks are shown to make the profiles more clear. (**B**) Sucrose gradient profiles of Δ*yjgA* strain and cells harboring YjgA mutations ΔNloop, D7A, D11A, D16A and E20A in the background of Δ*yjgA*. (C–F) interactions between GST-uL16 and His-YjgA (**C**), His-YjgA-ΔNloop (**D**), His-YjgA-DE-A (**E**) and His-YjgA-E23A (**F**) *in vitro*.

It is worth noting that the N-terminal loop of YjgA exhibited relatively lower conservation than the domain regions, yet it plays a pivotal role in 50S assembly (Figure 3A and E). To explore the functional significance of individual residues within the N-terminal loop, we introduced a series of single mutations and overexpressed them in Δ*yjgA* cells at 16°C followed by sucrose gradient analysis to assess their impact on 50S assembly (Figure 4A, B and Supplemental Figure S5A). Among these mutants, E6A, D21A, D22A, E23A and DE-A (all of the D and E residues were mutated to A) exhibit a significant increase in pre50S levels and show a higher peak than 30S peak (Figure 4A). The ratio of 50S/30S increases from 0.34 for YjgA-FL to 1.28–1.59 for these mutants, similar to the one in YjgA-*Δ*Nloop (Supplemental Figure S5A). Additionally, D7A, D11A, D16A and E20A show a slight increase in the peak of pre50S with the ratio of 50S/30S is 1.08–1.17 (Figure 4B and Supplemental Figure S5A). The mutant I17N also enhances the pre-50S peak, resulting in a 50S/30S ratio of 1.92 (Supplemental Figure S5A). Meanwhile, the other 14 mutations in the N-terminal loop show no effect on the subunit peaks (Supplemental Figure S5A). These results indicate that the negatively charged residues D and E play an important function in the 50S assembly process. By analyzing the amino acid composition within the N-terminal loop of YjgA, we found that these D and E residues, as well as the I17, are relatively conserved (Supplemental Figure S4A, labeled red triangle). The distribution of these D and E residues generates a negatively charged surface for the N-terminal loop (Supplemental Figure S5B). In consideration of the fact that the N-terminal loop of YjgA is near the ribosomal protein uL16, which exhibits a positively charged surface, we speculated that the N-terminal loop of YjgA may recruit or stabilize uL16 through polar interactions (Supplemental Figure S5B). The hydrophobic amino acid I17 might be used to determine the interaction site between YjgA and uL16 as well.

To confirm the interaction between YjgA and uL16, we performed a GST pull-down assay and noted that YjgA-FL effectively binds with uL16 *in vitro* (Figure 4C). However, upon deletion of the Nloop in YjgA, this interaction is abolished, which aligns with our earlier polysome profiling findings (Figures 4D and 3E). Furthermore, YjgA-DE-A, which disrupts 50S assembly, also exhibits a lack of interaction with uL16 (Figure 4A and E). Likewise, the YjgA-E23A mutation disrupts the interaction with uL16, which is in line with the findings from sucrose gradient profiling (Figure 4A and F). As a control, we chose a variant, YjgA-D15A, which has no impact on 50S assembly and maintains its ability to bind uL16 (Supplemental Figure S5A and C). This suggests that YjgA indeed relies on its Nloop to recruit uL16, aiding in the maturation of PTC.

Spot assay was also performed to check the effects of mutants on cell growth. The results showed that these mutants can be classified into four groups (Supplemental Figure S6). Group I showed a similar growth rate as YjgA-FL, e.g. W8A and E6A, indicating these mutants do not affect the growth rate, although they could affect the maturation of pre50S. This further suggests the perturbation of pre50S maturation does not significantly change the cell growth rate. Group II partially abolished the inhibitory effects of Nloop, including D16A, I17N, E20A, D21A and I24N. Group III (T2A, K3A, D22A and E23A) fully abolished the function of the Nloop in cell growth, showing similar rates as YjgA-ΔNloop, indicating these residues participate in the interactions of the Nloop with other factors besides uL16. Group IV, containing D7A, D11A and DE-A mutants, differs from those in the former groups. Specifically, D7A shows growth rates similar to Group I at 16°C but slower growth at 37°C; D11A exhibits slower growth at 16°C but normal growth at 37°C; and DE-A displays slower growth at 16°C akin to D11A, with minimal growth at 37°C. These three mutants, which do not follow a distinct pattern, have been categorized under Group IV. These mutants all showed a severe cell growth slowdown, probably they gained other unknown functions. Based on these results, we found the critical residues do not fully overlap between the pre50S maturation and cell growth, suggesting YjgA possesses some important and complicated functions besides its promotion on pre50S maturation. However, further research is needed to enhance our comprehensive understanding of YjgA.

### Structure comparison of pre50S_ΔNloop_ and pre50S_Δ_*_yjgA_*

**Figure 5.**
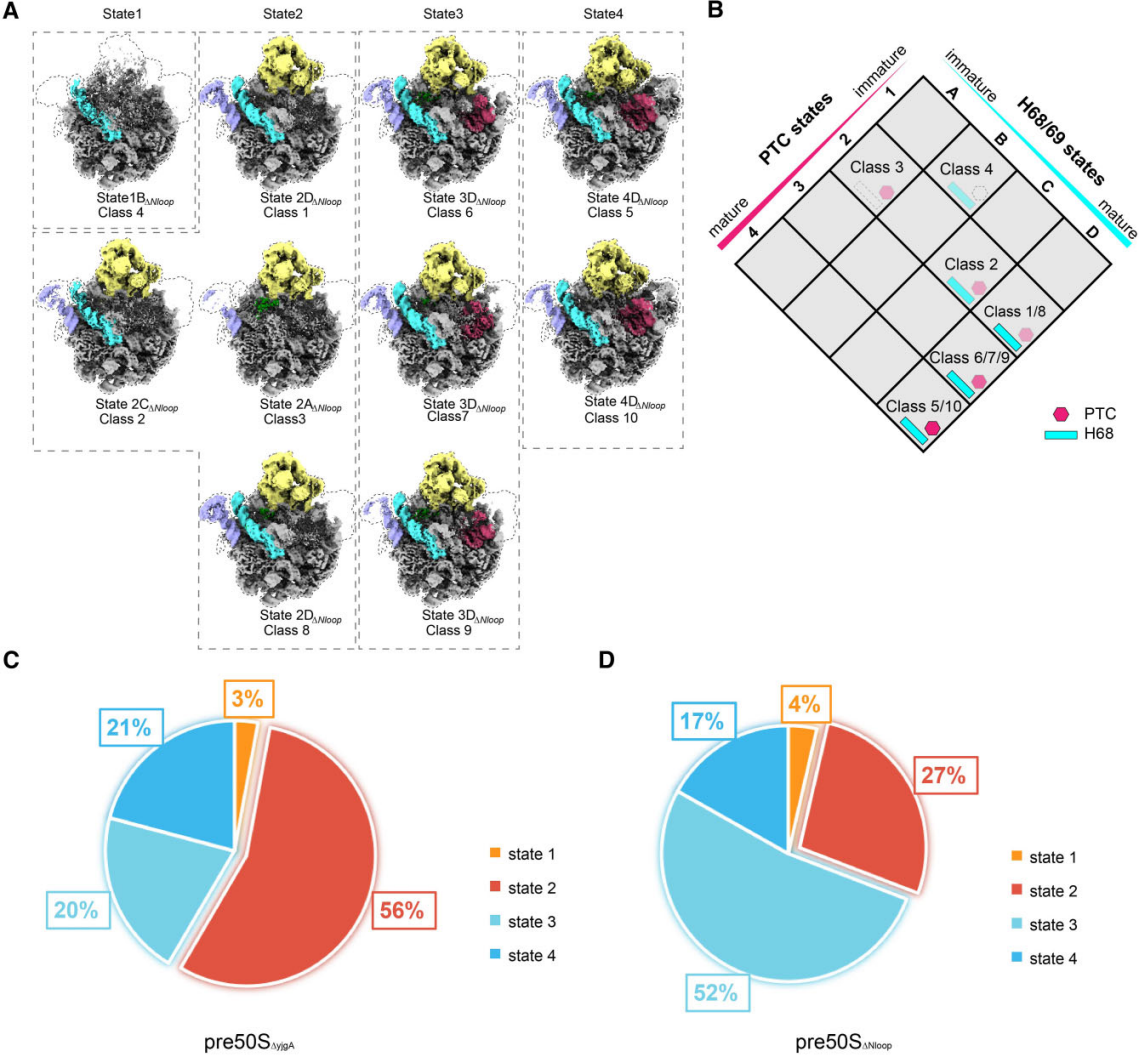
pre50S_ΔNloop_ shows multiple conformations. (**A**) Ten distinct density map groups were obtained by classification using cryoDRGN. Each image shows the average density map and reconstruction model of the corresponding group. These 10 maps represent 10 different structural states found in the pre50S_ΔNloop_. Purple is L1 stalk, yellow is CP, and pink is PTC. (**B**) Based on the maturation state of H68/69 and PTC, 11 density maps were classified into six distinct groups. These groups represent structural changes in pre50S at different stages of H68/69 and PTC maturation. States 1–4 and A–D correspond to pre50S intermediates with varying degrees of maturation. Blue represents the maturation of H68/69, and pink represents the maturation of PTC. (C, D) Distribution of occupancy for the four states of particles in cryoDRGN analysis of pre50S_Δ_*_yjgA_* (**C**) and pre50S_ΔNloop_ (**D**).

To further characterize the function of the N-terminal loop at the structural level, the peak of pre50S_ΔNloop_ from the sucrose gradient was then collected and applied for cryo-EM analysis as well (Figure 3E and Supplemental Figure S7). Same as the pre50S_Δ_*_yjgA_*, the overall structure of pre50S_ΔNloop_ also shows weak density for H89 in PTC region, indicating a markable restrain of YjgA-ΔNloop on PTC assembly (Supplemental Figure S7A compared with Figure 1C). The ribosomal proteins uL16, bL33, bL35, and bL36 are missing in this pre50S complex. And H38 and H89 only have weak density (Supplemental Figure S7A). For pre50S_ΔNloop_, 174, 541 particles were grouped into 500 volumes using kmeans clustering with K = 500, which ensures that each volume contains enough particles (Supplemental Figure S8). With hierarchical clustering, the particles of pre50S_ΔNloop_ were also grouped into 10 classes, which were categorized into 4 states as in pre50S_Δ_*_yjgA_* (Figure 5A and B). The PTC regions of these four states are consistent with the four classes observed in pre50S_Δ_*_yjgA_*, and there is also a swinging trend in the H68/69 region (Figure 5A and B).

In addition, the number of particles for each state of pre50S_Δ_*_yjgA_* and pre50S_ΔNloop_ were counted separately (Figure 5C and D). Compared to pre50S_Δ_*_yjgA_*, the reintroduction of YjgA-ΔNloop changed the ratio of states 2 and 3 but kept state 1 and state 4 similar in the two pre50S intermediates. When YjgA was not present, the majority state was state 2 (56%) and the second was state 3 (20%) (Figure 5C). As described above, state 2 has very few densities for the PTC helices, and state 3 had most of the helices folded except H89 (Figure 2D). When YjgA-ΔNloop was overexpressed, state 2 decreased to 27%, and state 3 increased to 52% (Figure 5D). These results suggest that YjgA-Nloop still has the function of undocking the H68/69, thus providing space for PTC maturation, so that state 2 with unmatured PTC decreased from 56% to 27%. Due to the deletion of the N-terminal loop, the binding of uL16 is affected and results in a flexible H89 in state 3. This again demonstrates that the N-terminal loop is critical for promoting state 3 to state 4, where the uL16 and H89 are stabilized and generate a fully mature PTC.

**Figure 6.**
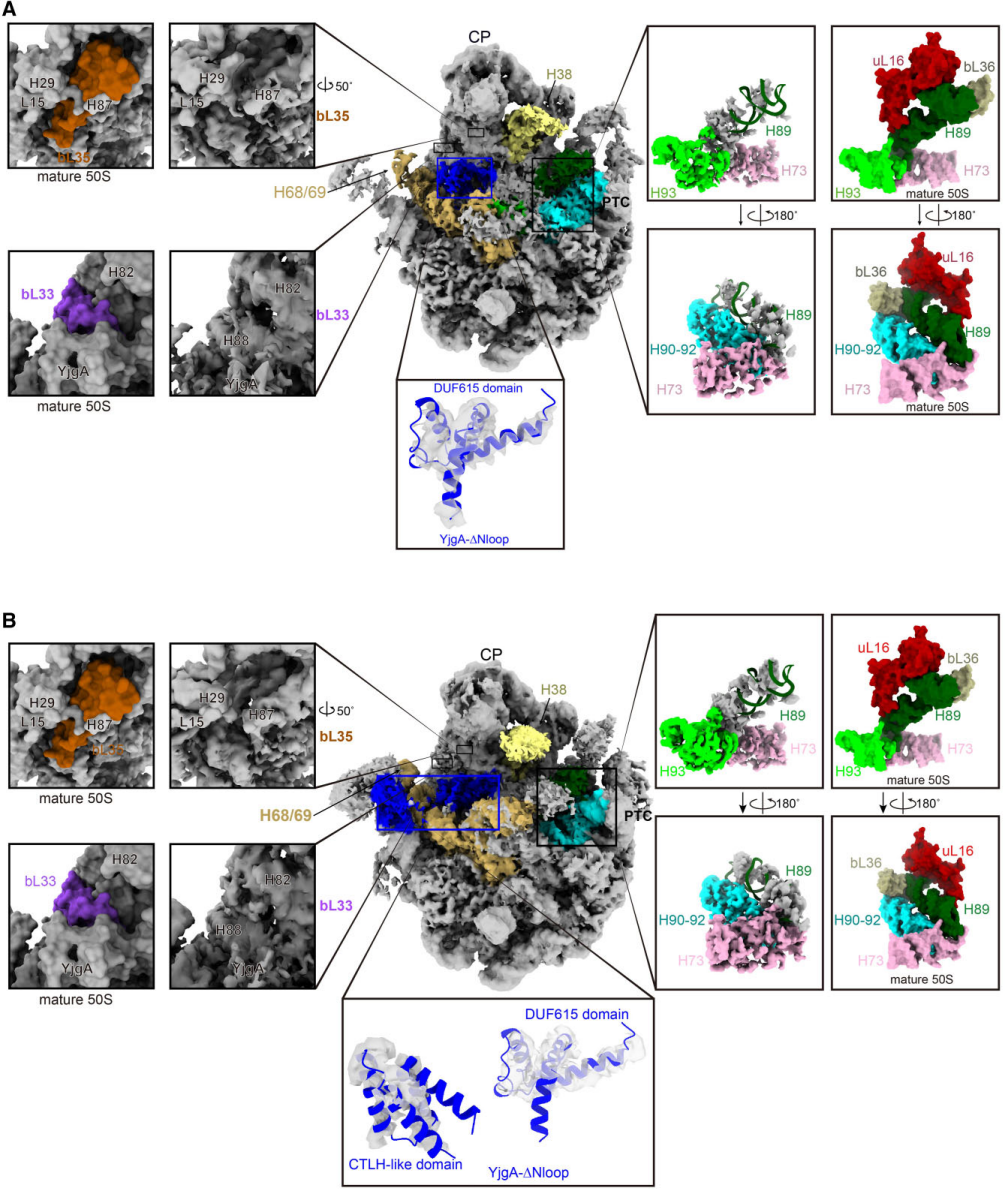
Two conformations of YjgA in pre50S_ΔNloop_. (A, B) the overall structure of YjgA-ΔNloop on pre50S showing the binding of either the DUF615 domain alone (**A**) or with the CTLH-like domain (**B**). Density of the YjgA-ΔNloop protein is shown in gray surface below the overall structure in both figures. Details of the PTC, bL33 and bL35 are zoomed up to represent the density occupancy of H89, uL16, bL33, bL35 and bL36. The models of the PTC, bL33 and bL35 in a mature 50S (PDB: 7K00) are shown in the surface as a reference.

In contrast to pre50S_Δ_*_yjgA_*, further analysis using the k-means clustering method in cryoDRGN identified two reconstructions that contained YjgA-ΔNloop protein (Figure 6A and B). The YjgA-ΔNloop shows two conformations, with one only having its DUF615 domain identified (Figure 6A) and the other one with both the DUF615 and CTLH-like domain observed (Figure 6B). Similar to the binding of WT-YjgA, both conformations of YjgA-ΔNloop interact with H88 (6,7,18). The one showing a CTLH-like domain is also associated with L1-stalk, the same conformation as observed previously in a complex with pre50S-ObgE (7) (Figure 6B). By comparing the two structures, we observed that the interactions involved in the CTLH-like domain lead to a minor displacement of the L1-stalk towards the CP region (Figure 6B). Both structures exhibit well-formed H68/H69 and H90-93 regions. However, neither of them displays good electron density for uL16 or H89 (Figure 6A and B). These results indicate that the deletion of YjgA’s N-terminal loop influences the binding of uL16, thus preventing the stabilization of H89.

### Model of YjgA-dependent 50S maturation

**Figure 7.**
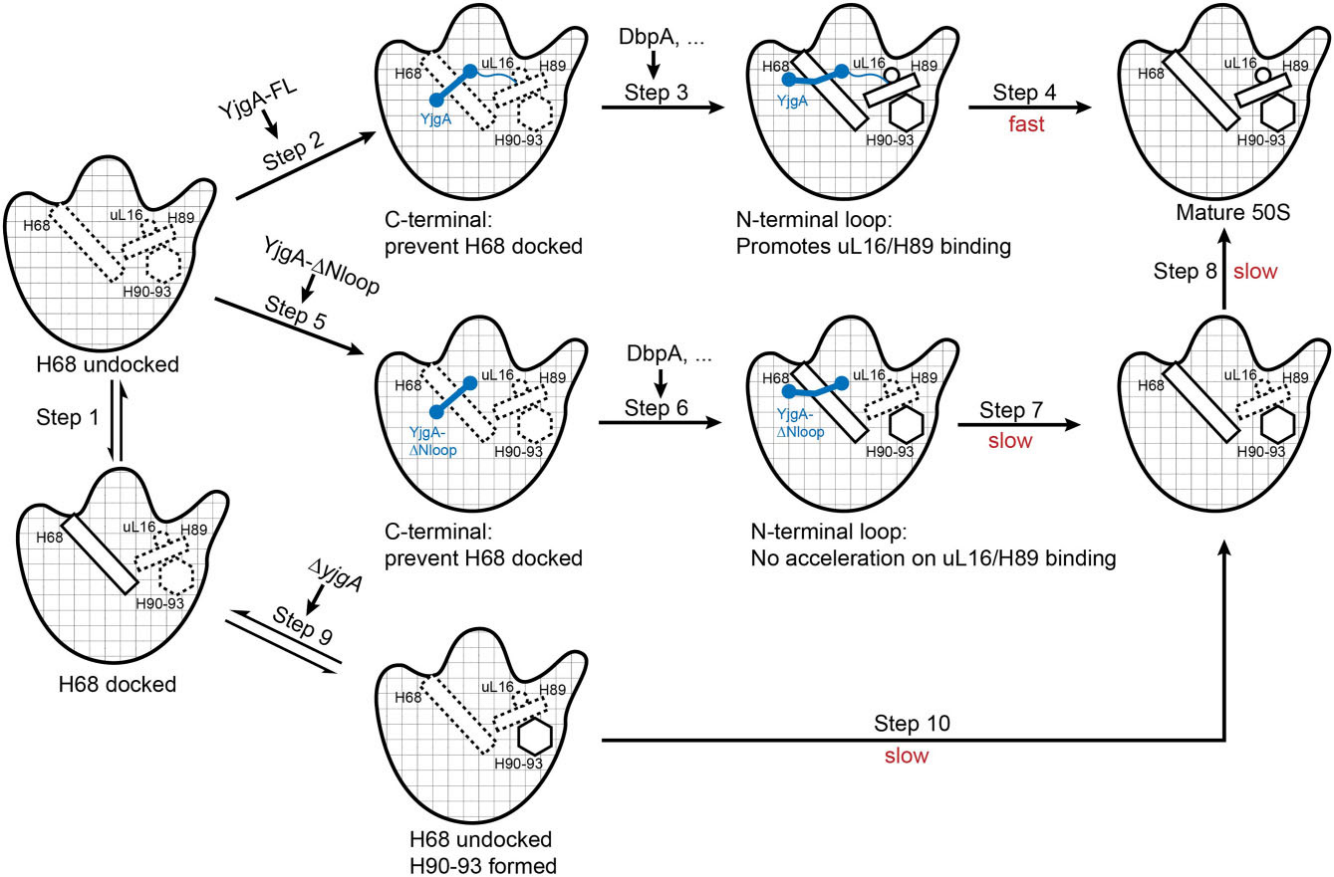
Model of YjgA-mediated 50S maturation pathways. When full-length YjgA protein is present, it binds to the base of CP through its DUF615 domain, preventing the docking of H68/69, generating a more flexible connector H71 and providing space for PTC folding. The N-terminal loop of YjgA also promotes the binding of uL16 and stabilizes H89, accelerating the maturation of PTC. However, if YjgA is removed or YjgA-ΔNloop is expressed instead, the folding of PTC is affected, resulting in unstable uL16 and H89. These findings highlight the importance of different structure elements of YjgA protein in 50S maturation.

Based on the facts we observed in this study that both the N-terminal part and C-terminal part participate in the assembly of the PTC region, we proposed a comprehensive model of 50S maturation related to YjgA (Figure 7). Firstly, under normal conditions, the pre50S particles will go to a state where most of the rRNA fold well except the PTC region and the long H68/69 helix. In this situation, the paired H68 could dock to the base of CP and L1-stalk spontaneously, and H68 exists in a balanced state between the docked and undocked conformations (step 1). When the PTC is ready to be folded, if full-length YjgA is present, the protein will bind to the base of CP through the interactions between its DUF615 domain and H88. In the equilibrated state that H68 undocks, the CTLH-like domain will associate with the base of the L1-stalk (step 2). This binding process will prevent the docking of H68, generating a more flexible connector H71 and providing enough space for PTC folding (6,7) (step 3). After the H90-93 in PTC is mature, H68 tends to be docked and YjgA stays in a bent conformation. In this pathway, the N-terminal loop could promote the binding of uL16 and stabilizing H89, hence accelerating the final maturation of PTC (step 4).

If YjgA-ΔNloop is expressed instead, the protein will bind to the same place as full-length YjgA to prevent H68 docking and provide space for PTC maturation (steps 5 and 6). However, the assistance of uL16 binding and H89 folding is omitted, resulting in a PTC with uL16 missing and H89 unstable (step 7). In this case, other assembly factors would assist the integration of uL16 and finally generate a mature PTC (step 8).

In the case of Δ*yjgA*, PTC will fold with the help of DbpA and some other assembly factors in the status that H68 undocked (step 9). Since there is no YjgA protein in the system, uL16 and H89 are also unstable, as observed in YjgA-ΔNloop (step 10). This model points out the importance of the bifunctional small protein YjgA in PTC maturation, providing structural insights into the late pre50S intermediates.

## Discussion

Biogenesis of ribosomes from bacteria to humans requires lots of assembly factors for accuracy and efficiency. However, their specific roles in the assembly process remain poorly understood. In the present study, we characterized the structures of immature pre50S_Δ_*_yjgA_* and pre50S_ΔNloop_ particles. Comparison of these structures with mature 50S showed that the most intriguing deviation is the disordered status of the PTC and the high conformational flexibility of H68/69. Combine with cryo-EM analysis and functional experiments, we suggest that YjgA can promote PTC maturation by modulating the docking of H68 and maintaining the stability of H89 with the help of uL16.

YjgA has been found to have two conformations on the pre50S particles connecting the base of CP to the L1-stalk (6,7). The extended conformation is exclusive with the docking of H68 and the bent conformation binds with the L1 stalk on top of the docked H68 (6,7). H68 is a component of the B2b and B7a intersubunit bridges, which mediate interactions of the small and large ribosomal subunits with the incoming tRNA (41). The flexible nature of H68 has also been reported in a recent study of *S. aureus* (42) and *M. smegmatis* 50S subunit (43). The docking of H68 was shown to be rate-limiting during the late-stage assembly of the 50S subunit when studying EngA and RbgA, and the flexible conformation of H68 makes the 50S subunit incompatible for associating with the 30S subunit (8,43,44). Thus, dissociation of YjgA is suggested to be a signal of maturation, releasing the mature large subunit to bind the small subunit (6). However, when the *yjgA* gene was deleted, we obtained eight pre50S_Δ_*_yjgA_* structures showing different levels of density for H68 and PTC (Figure 2C), suggesting that YjgA is not only an intelligencer but also plays functions in the assembling of H68 and PTC.

One of the functions might be recruiting or stabilizing uL16, which is located between H38 and H89. It was reported that uL16 is important for the proper placement of tRNA in the A-site (45), and it could stabilize H89 and H38 through its α-helices and the flat-concave surface of its β-sheet, respectively (46–48). Entry of uL16 is one of the last maturation events and has a significant effect on the placement of the rRNA helices forming the functional sites (3). Our structures show that the deletion of YjgA or its N-terminal loop could destabilize the binding of uL16 based on the observation of weak density for uL16, H38 and H89. The accumulation of pre50S peak when expressing mutants for the negatively charged residues further confirmed the importance of this N-terminal loop. The pull-down assay between YjgA and its mutants with uL16 further confirmed that YjgA indeed interact with uL16 through its Nloop. The assembly factor ObgE could also affect the bindng of uL16, but in a way that interrupts the folding of H89 (7). We should note that the lack of uL16 on 50S particles accumulated ObgE, probably by impairing the GTPase activity of ObgE (7). However, in our structures, no density was found to correspond to ObgE. One reason could be that the function of YjgA in stabilizing uL16 is limited, not a major and irreplaceable role. Xiaoxiao et al. proposed that the species-specific C-terminal extension of EngA could have potential interactions with the PTC helices and uL16 in its proximity (8). RbgA is another GTPase that can affect uL16 binding, and its activity is, in turn, dependent on uL16, just like in ObgE (3).

The other function of YjgA would be undocking H68 and providing enough space for PTC folding. H68/69 forms a long helix near PTC that could have steric hindrance with the folding intermediates of PTC. The connecting point between H68/H69 and PTC is H71. During *in vitro* assembly, H71 aids in the proper folding of H90–H93 within the PTC (22). The PTC and H68/69 can affect each other through H71, which is precisely the impact caused by their spatial structure. Previous research has observed a coordination between the folding of H68 and PTC in the Δ*rrmj* (13). Through the *in vitro* reconstruction pathways, PTC is the last element to be matured. Assembly factors such as ObgE, SrmB, EngA and deaD have been identified to impact the maturation of the PTC *in vivo* (4,7,8,19). Among them, EngA is believed to interact with the PTC through its positively charged amino acid residues at the C-terminus, and ObgE could help the folding of H89 (7,8). As shown in pre50S_ΔNloop_ structures, the density of YjgA-ΔNloop and H68 still exhibited mutually exclusive patterns. Pre50S_ΔNloop_ has more particles in the PTC-folded states than pre50S_Δ_*_yjgA_* suggesting a correlation between H68/69 docking and PTC maturation. However, ribosome maturation is complex, involving multiple assembly pathways, so H68 undocking might not be the sole determinant of PTC maturation.

Although YjgA does not fall under the category of RNA-modifying enzymes or RNA-remodeling proteins, its pivotal role in 50S subunit maturation underscores the significance of this relatively understudied protein. The involvement of numerous other assembly factors in ribosome maturation suggests that these proteins may not be indispensable for biogenesis but are vital for maintaining normal cellular function. Our finding that YjgA possesses dual roles in promoting PTC maturation provided insights for constructing a comprehensive pathway of ribosome biogenesis.

## Supplementary Material

gkae469_Supplemental_File

## Data Availability

Cryo-EM maps of pre50S_Δ_*_yjgA_* and pre50S_ΔNloop_ have been deposited in the Electron Microscopy Data Bank (EMDB) with accession codes EMD-36330 and EMD-36329, respectively.
