# Supplementary material for: YjgA plays dual roles in enhancing PTC maturation
Source: Nucleic Acids Res. 2024 Jun 6;52(13):7947–60. doi: 10.1093/nar/gkae469 (PMC11260487; doi:10.1093/nar/gkae469)
Supplement: gkae469_Supplemental_File [file gkae469_supplemental_file.pdf]

SUPPLEMENTARY DATA

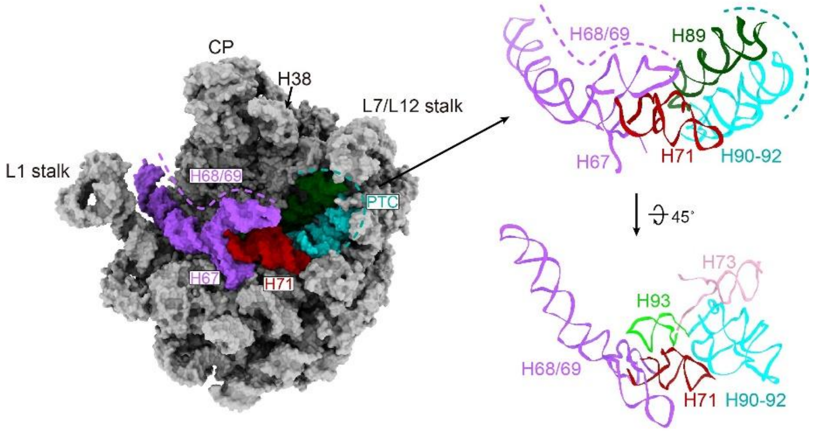

Supplemental Figure 1. The relationship of H68/69 and PTC. Overall structure of mature 50S (7K00) shown in surface. The H67/68/69 (purple), H71(red) and PTC components (other colors) are zoomed up to represent the relationship of H68/69 and PTC shown in cartoon.

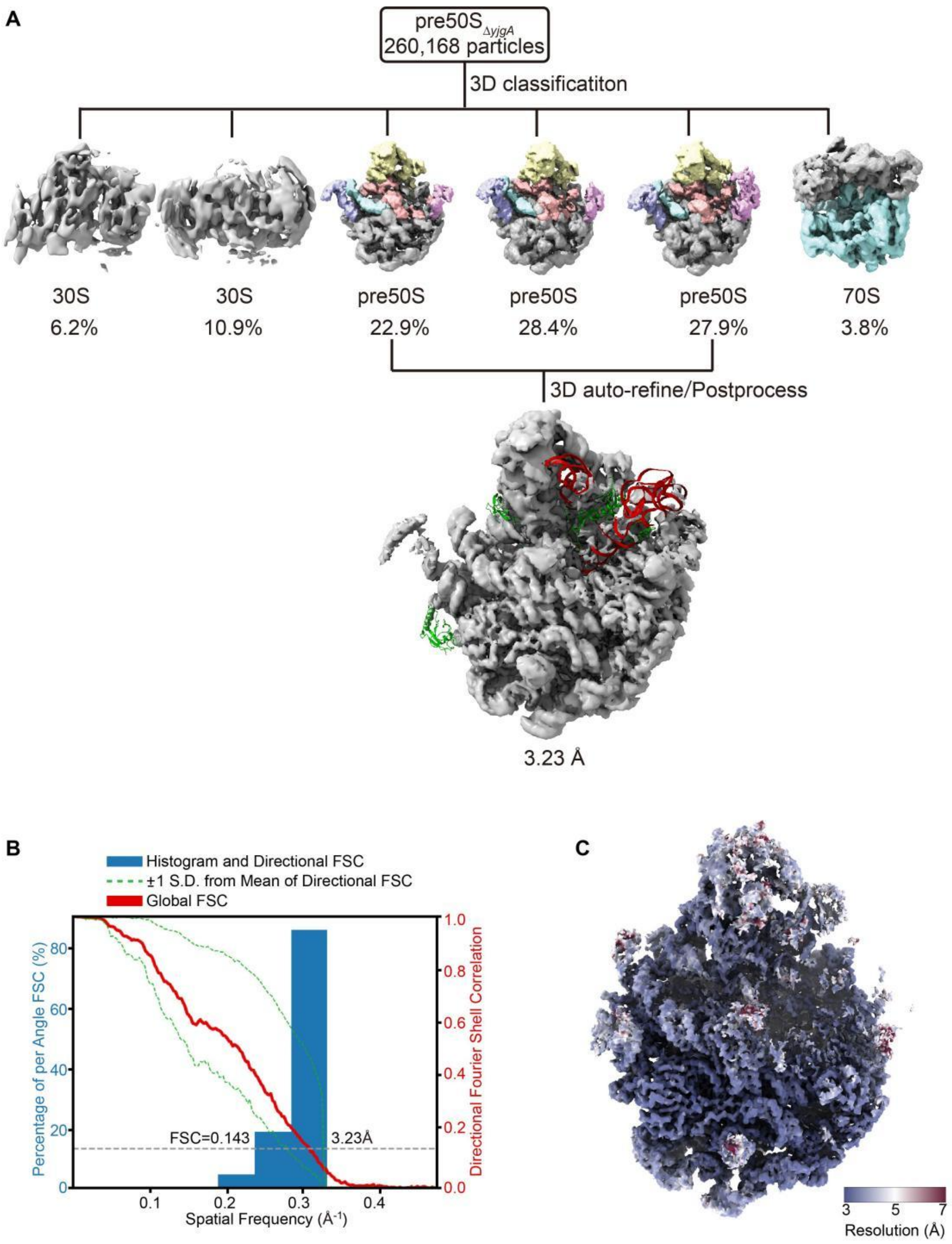

Supplemental Figure 2. Data process of pre50S$_{\Delta yjgA}$ and density evaluations. A, Workflow for data process of pre50S$_{\Delta yjgA}$ in Relion. A total of 260, 168 particles were selected from several rounds of 2D and 3D classifications for discarding non-ribosomal particles. These particles were then submitted to 3D classification to isolate the different subunit/conformation of ribosomes. A final reconstruction of 3.23 Å was obtained for the pre50S intermediates. **B,** Gold-standard FSC curves for the electron microscopy map of pre50S$_{\Delta yjgA}$. Resolution is demarcated using the FSC=0.143 criterion. **C,** Local-resolution-filtered map which was colored according to local resolution.

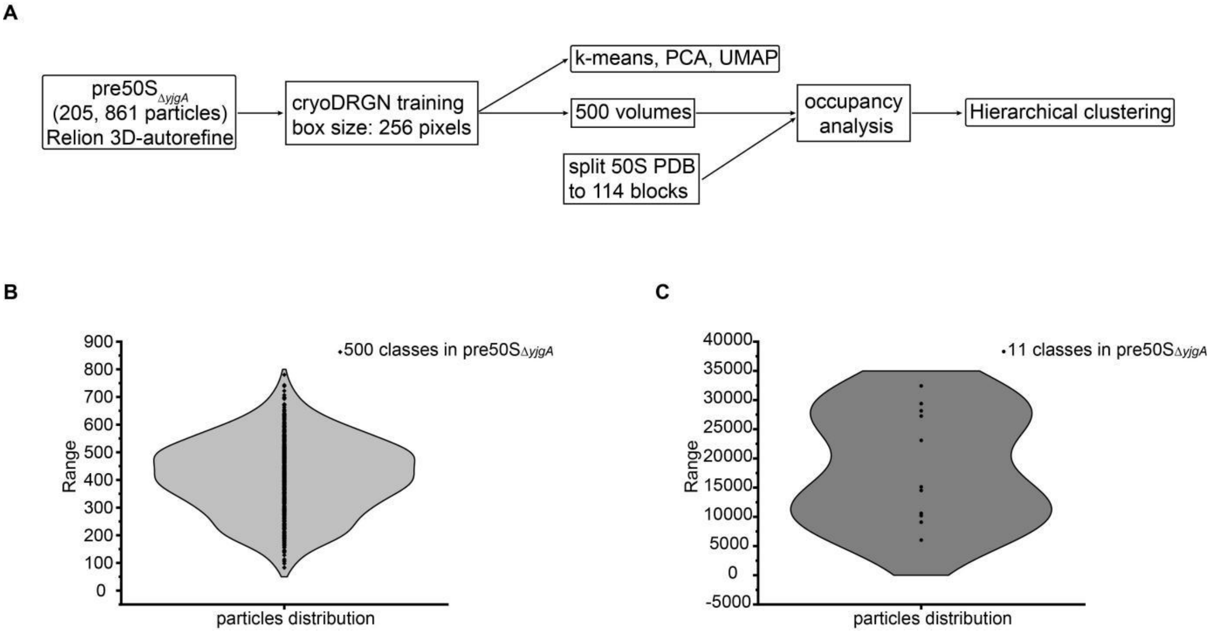

Supplemental Figure 3. Data process of pre50S$_{\Delta yjgA}$ in cryoDRGN. A, workflow of data processing in cryoDRGN for pre50S$_{\Delta yjgA}$ particles. **B**, distribution of the particle numbers for the five hundred volumes used by cryoDRGN. **C**, distribution of the particle numbers for the final ten classes.

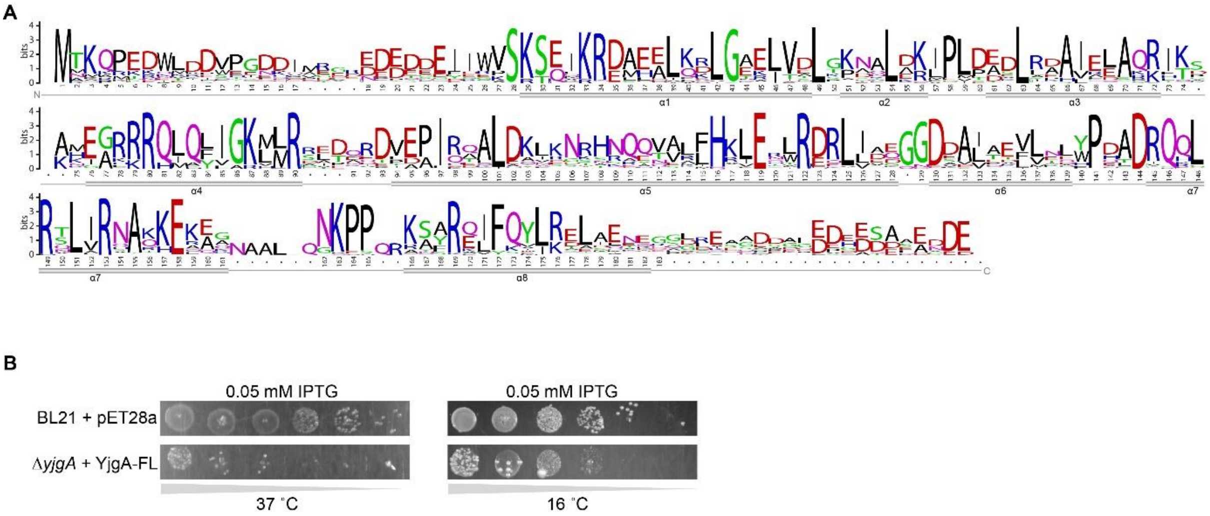

Supplemental Figure 4. YjgA is highly conserved in bacteria. A, The 143 reviewed YjgA sequences were collected from UniProt and submitted for alignment with ClusterW. Conservation of YjgA is shown in WebLogo, indicating the amino acids preferences. Amino acids observed in increasing pre50S peaks are labeled in red triangles (▲). **B,** Spot assay of BL21 strain harboring empty vector (pET28a) and Δ*yjgA* harboring YjgA-FL at 37 ˚C and 16 ˚C.

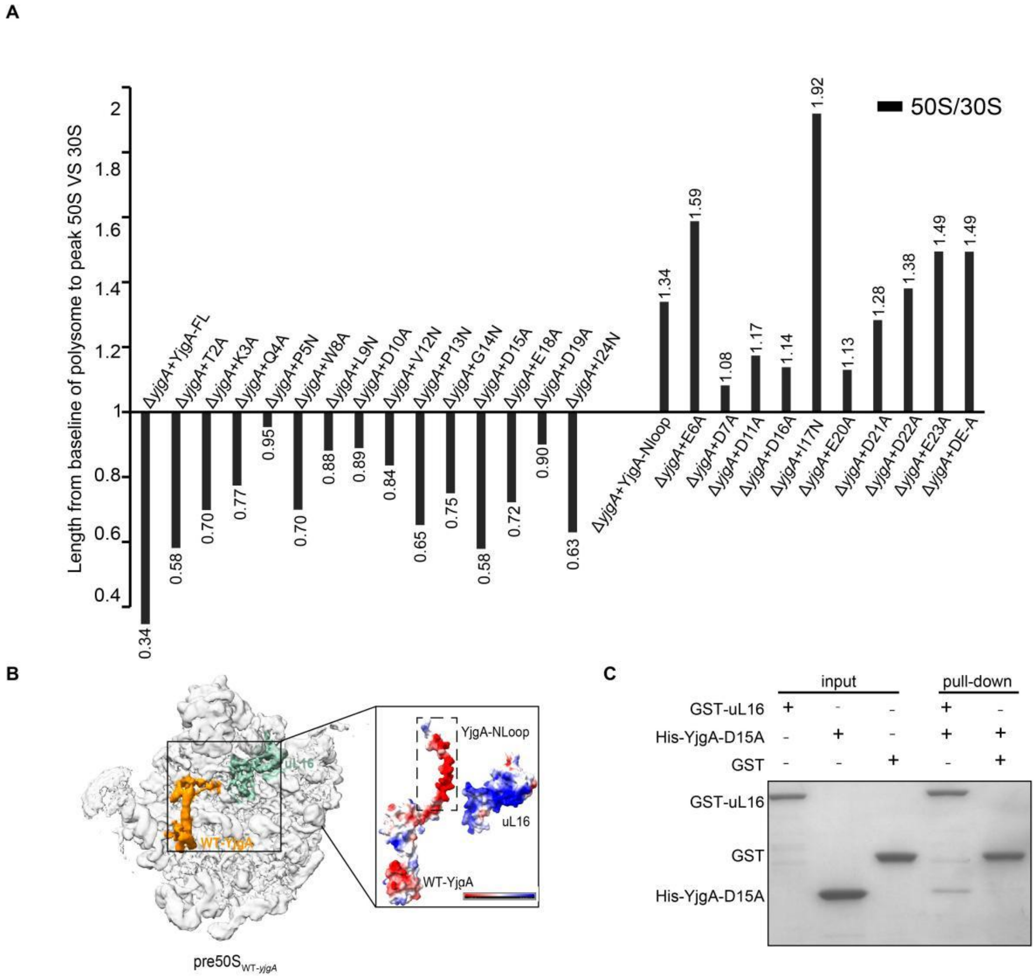

Supplemental Figure 5. Mutation of the negatively charged residues in the N-terminal loop of YjgA impair 50S assembly. A, The ratio of 50S/30S was calculated from the sucrose gradient profiles that expressed each mutant in the background of Δ*yjgA*. **B,** N-terminal loop of YjgA may recruit or stabilize uL16 through polar interactions. **C,** Interactions of GST-uL16 with His-YjgA-D15A *in vitro*.

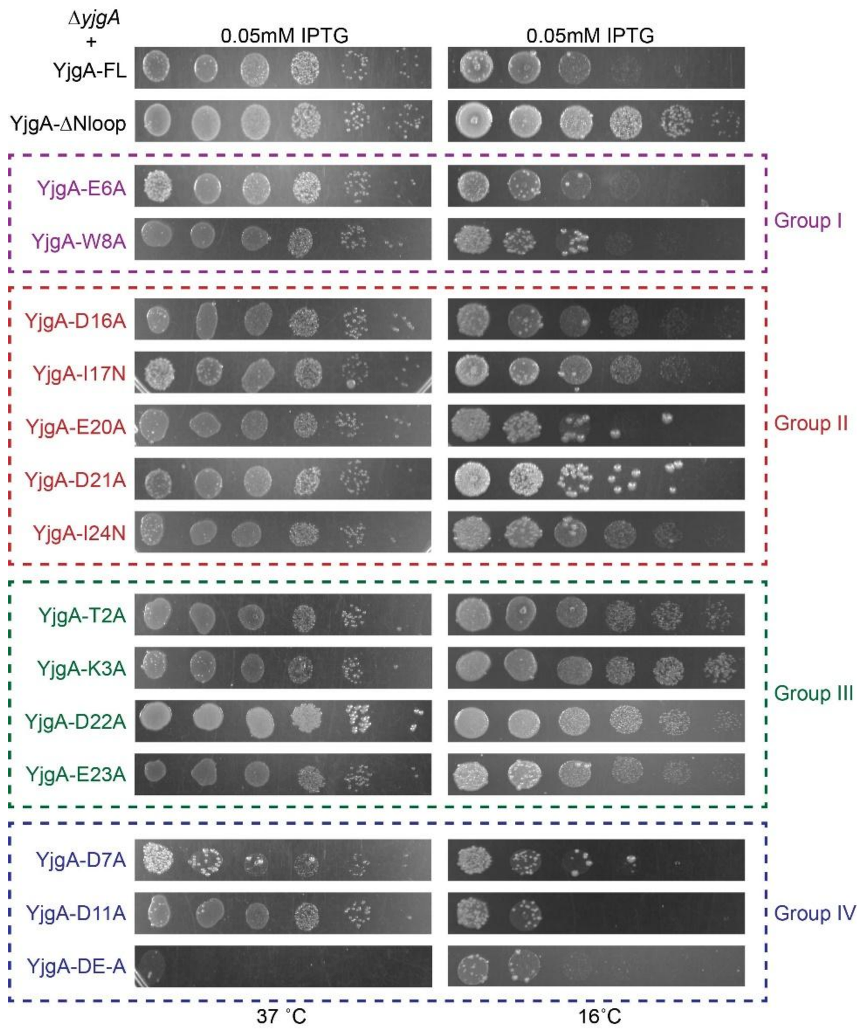

Supplemental Figure 6. Some of the residues in the N-terminal loop of YjgA are important for cell growth. Spot assay of Δ*yjgA* harboring YjgA-FL and YjgA mutant at 37 ˚C and 16 ˚C. Based on the growth rate, these residues are classified into four groups (I, II, III and IV).

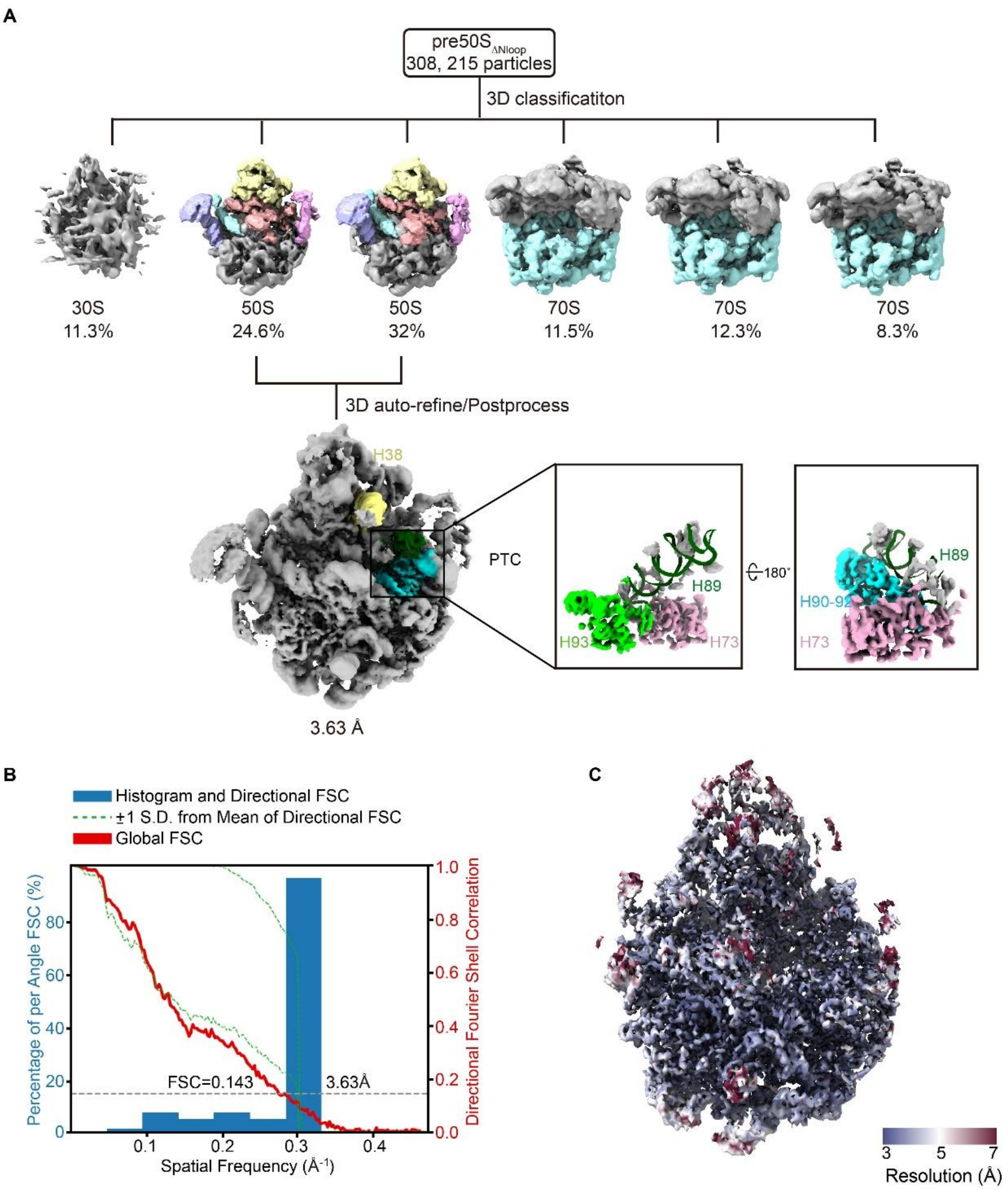

Supplemental Figure 7. Data process of pre-50S$_{\Delta Nloop}$ and density evaluations. A, Workflow for data process of pre-50S$_{\Delta Nloop}$ in Relion. Details of the PTC are zoomed up to represent the density occupancy of H89 H90-92 and H93. **B,** Gold-standard FSC curves for the electron microscopy map of pre-50S$_{\Delta Nloop}$. Resolution is demarcated using the FSC=0.143 criterion. **C,** Local-resolution-filtered map which is colored according to local resolution.

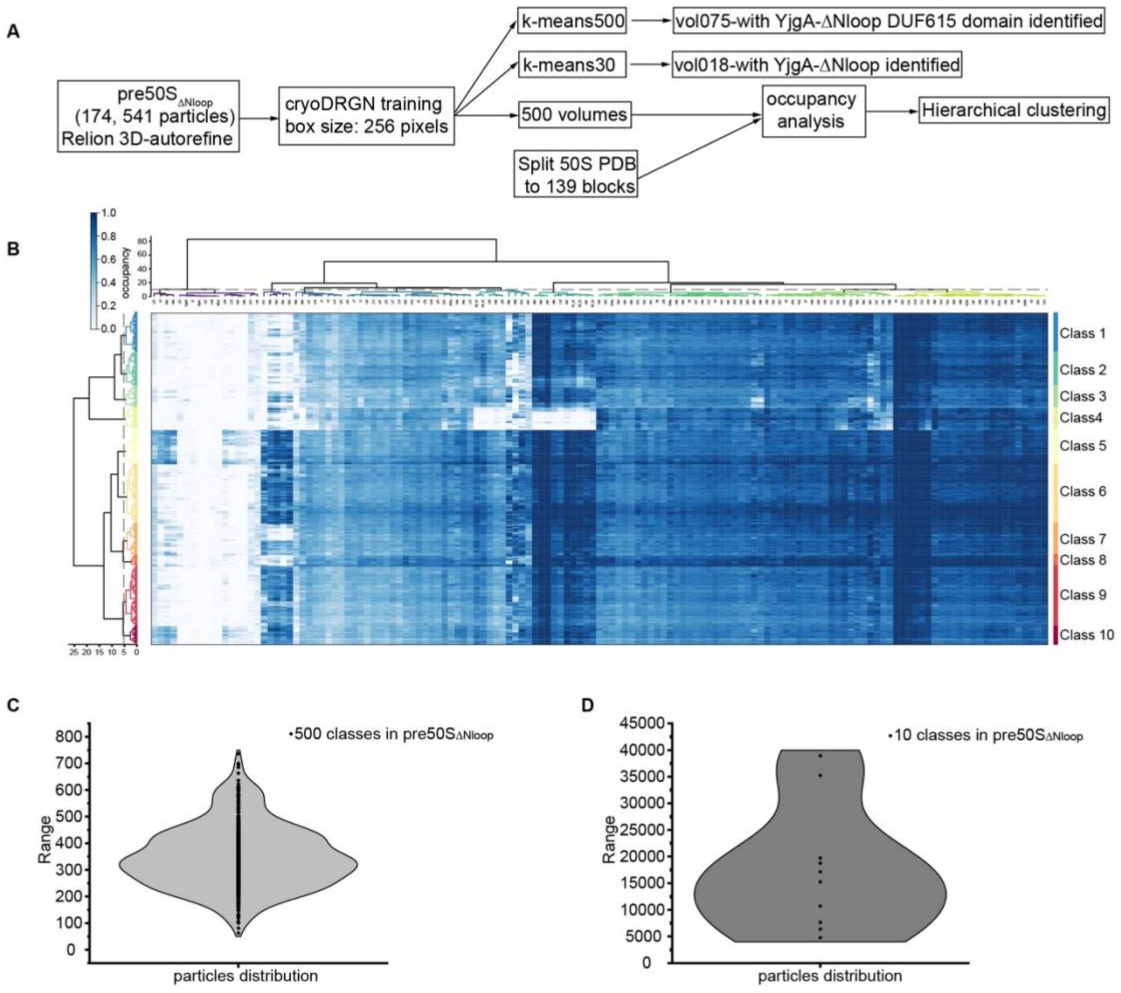

Supplemental Figure 8. Pre50S$_{\Delta Nloop}$ exhibits multiple conformations. **A**, workflow of data processing in cryoDRGN for pre50S$_{\Delta Nloop}$ particles. **B**, five hundred volumes of pre-50S$_{\Delta Nloop}$ were segmented using cryoDRGN and a heatmap was generated based on their similarities. **C**, distribution of the particle numbers for the five hundred volumes used by cryoDRGN. **D**, distribution of the particle numbers for the final ten classes.
